# The Bacteriological Diagnosis of Diphtheria

**Published:** 1898-10-15

**Authors:** J. O. Symes

**Affiliations:** Bacteriologist, Bristol Royal Infirmary.


### Medical Progress and Hospital Clinics.

### THE BACTERIOLOGICAL DIAGNOSIS OF DIPHTHERIA.

By J. O. Symes, M.D., D.P.H., Bacteriologist, Bristol Royal Infirmary.

The bacillus of diphtheria was first described by Klebs in 1883, and was isolated and cultivated by Löffler in the following year. Later the investigations of Roux, Yersin, and Sidney Martin showed that the characteristic symptoms of the disease were caused by a specific toxin elaborated by the organism, and which was capable of separation and examination in vitrio.

The organism was described as being polymorphic, there being three well-recognised types: (*a*) Irregularly staining club forms; (*b*) dumb-bell forms, with darkly staining swollen ends; (*c*) straight or slightly curved rods, swollen in the centre and tapering at the ends.

Both of the two last varieties (*b* and *c*) are subject to considerable modifications with regard to length; and, indeed, there has been an attempt to classify diphtheria bacilli into long and short varieties. The members of these three groups agree with one another in all essential diagnostic points; thus they all form small, circular, opaque, whitish colonies on blood-serum or agar within twenty-four hours; whilst in bouillon they produce a powdery sediment, the medium becoming first acid and then again alkaline. They are all pathogenic to certain animals, such as the rabbit. There can be no doubt that these are but modified forms of the micro-organism described by Klebs and Löffler. In 1887, however, Löffler described a short bacillus closely resembling the diphtheria bacillus, both in methods of growth and staining reactions, but differing in that it did not form acid in bouillon, nor was it pathogenic to animals. Von Hoffman[1] described a similar, or probably the same, organism in 1888. These short bacilli may be found, together with the Klebs-Löffler bacillus, in undoubted cases of diphtheria; they may occur alone in simple anginas, or may be found in apparently healthy throats. They have been called pseudo - diphtheria bacilli. Biggs,[2] Cobbett and Phillips,[3] Parke and Beebe,[4] and Peters have all described bacilli (generally short bacilli) which closely resemble the diphtheria and pseudo - diphtheria organisms. Biggs found two varieties of the pseudo-diphtheria bacillus, one of which produces an acid reaction in bouillon, whilst the other does not.

It is then abundantly evident that there are a large number of micro-organisms found in both healthy and diseased tissues which can either be distinguished only with difficulty, or which cannot be distinguished at all from the specific organism of diphtheria. The question of the relation of these bacteria to the disease is one of great interest and practical importance. The micro-organisms which lead to confusion in the diagnosis of diphtheria are then—(1) Short forms of the Klebs-Löffler bacillus; (2) pseudo-diphtheria bacilli, some producing an acid, others an alkaline reaction; (3) unclassified forms more or less resembling the Klebs-Löffler bacillus. We may at once dismiss the members of the third class, of which Neisser's Xerosis bacillus may be taken as an example. Not only are they non-virulent to animals, but the employment of various culture methods and staining reactions will reveal marked morphological differences between these organisms and the true diphtheria bacillus.

With regard to the short forms of diphtheria bacilli, it is still uncertain whether these are to be regarded as distinct varieties or as modifications of the long bacilli. Peters,[5] in a series of experiments extending over two years, failed to convert the short into the long variety, the only change he noted being a complete, or in some cases a partial, loss of virulence.

Most workers who have had to deal with a large number of diphtheria cultures will however, we think, be inclined to regard the short virulent diphtheria bacillus as a modification of the longer type. The two varieties are quite commonly found in the same culture, and, during the convalescence of a case in which typical long bacilli have been found, it will be noticed that these gradually become replaced by the short, which in their turn gradually disappear. We are so familiar with the variations of the organism in vitrio that the occurrence of even more marked changes whilst in the human body is not surprising.

The question of the relation of the pseudo-diphtheria bacillus to the disease is a much more difficult one. These organisms are to be found in normal throats, in simple anginas, and in the angina of scarlet fever. If mistaken for the short diphtheria bacillus, the consequences may be very serious; thus the patient may be isolated unnecessarily, may be associated with diphtheria patients, or may be detained for weeks or months as an infected person.

The pseudo-bacillus, or, as it is sometimes called, Hoffman's bacillus, has a close resemblance to the Klebs-Löffler organisms. It is, however, shorter, the ends are not swollen, it grows better on agar, and some varieties at least are not capable of forming acid in bouillon. The most important distinction, however, is that the pseudo-bacillus is not pathogenic to animals. Quite recently Neisser[6] has pointed out still another method of distinguishing between Von Hoffman's and Löffler's bacilli. Film preparations are stained for three seconds in a solution consisting of 1 grain of methylene blue dissolved in 20 cc. of 96 per cent. alcohol, 950 ccs. of distilled water, and 50 ccs. of glacial acetic acid; they are then washed and stained for five seconds in a solution made by dissolving 2 grains of benzoin in 1,000 ccs. of distilled water and filtering. The Klebs-Löffler bacilli are stained brown with two or three granules of dark blue or black. Von Hoffman's pseudo-bacillus does not show this granulation. Hewlett and Knight,[7] however, have published a most important series of observations which would point to the conclusion that the pseudo-bacilli are but modified forms of the virulent germ. In genuine diphtheritic anginas, they found the pseudo-bacilli to gradually replace the Klebs-Löffler bacilli as the case progressed to convalescence, and between the two varieties they found a series of organisms which formed a connecting-chain. They were also able by careful heating to convert typical virulent Klebs-Löffler bacilli into atypical non-virulent pseudo-bacilli, and on the other hand, by cultivation on various media, by incubation, and by passage through an animal, to modify the pseudo-bacillus into the virulent diphtheria organism.

Their experience is diametrically opposed to that of such observers as Von Hoffman, Parke and Beebe, Biggs, and Peters, none of whom regard the pseudo-bacillus as in any way related to the Klebs-Löffler organisms, and none of whom succeeded in converting the one into the other.

As the matter stands at present we must wait for further evidence before we can definitely decide upon the relationship of these bacteria to one another.

We have shown that there exists great diversity of opinion as to what varieties should and what should not be regarded as diphtheria bacilli. In actual practice, however, there is a great tendency to call any short bacillus, more or less conforming to the types described, a diptheritic bacillus, and to notify, isolate, and treat the case as one of diphtheria. Probably this is the safer course, and it is only in those cases where the convalescence is prolonged that any injustice and harm is done. Cultures are taken from the throat of a child supposed to be recovering from an attack of diphtheria, and the report comes back that diphtheria bacilli are still present. This condition may continue for weeks or months, the child being detained and treated as an invalid because short bacilli still occur in the cultures. It is in cases such as these that special investigation of the organism is called for. Its mode of growth and microscopical appearances, its power of forming acid in bouillon culture media, its reaction to Neisser's stains, and, if possible, its virulence, should all be tested, and should the organism be found to conform to the pseudo type, then the isolation restrictions may be relaxed, it being obviously absurd to guard against a bacillus which is so frequently found to be an occupant of healthy throats. Should, however, the observations of Hewlett and Knight be confirmed, and it be found that the pseudo-bacillus may become virulent under certain circumstances, then isolation will have to be prolonged, as at present, until short forms disappear. At present the evidence in favour of the pathogenic powers of the pseudo-bacillus can in no way be regarded as conclusive.

1 Wien. Med. Wohnschr. (1888), Nos. 3 and 4. 2 Scientific Bulletin, No. 1, Health Report, City of New York. 3 Journal of Pathology and Bacteriology, iv., No. 2, 1896. 4 New York Med. Rec., lxvi., 1894. 5 Journal Path. and Bacteriol., iv., No. 2, 1896, page 181. 6 Zeitschrif. f. Hyg., xxiv., 1897, p. 443, 7 Transact. Brit. Instit. Prevent. Medic., first series.

